# Serendipitous preparation of *fac*-(aceto­nitrile-κ*N*)tri­chlorido­[(1,2,5,6-η)-cyclo­octa-1,5-diene]iridium(III)

**DOI:** 10.1107/S2056989015004855

**Published:** 2015-03-14

**Authors:** David M. Morris, Joseph S. Merola

**Affiliations:** a Department of Chemistry, Virginia Tech, Blacksburg, VA 24061, USA

**Keywords:** crystal structure, iridium, cyclo­octa­diene, aceto­nitrile

## Abstract

A reaction between [(COD)IrCl]_2_ (COD is cyclo­octa-1,5-diene), HCl and indene failed to provide the hoped for chlorido­indenyliridium dimer, but instead produced the title compound, [IrCl_3_(CH_3_CN)(C_8_H_12_)], which is an octa­hedral complex of iridium(III) with a chelating cyclo­octa-1,5-diene ligand, three chloride ligands in a *fac* arrangement, and one aceto­nitrile ligand. Attempts to devise a rational synthesis for the title compound were unsuccessful.

## Abstract 2

An octa­hedral complex of iridium(III) with a chelating cyclo­octa-1,5-diene ligand, a facial arrangement of three chloride ligands and one aceto­nitrile ligand was isolated serendipitously from an attempted reaction between indene, the chlorido­cyclo­octa­dieneiridium dimer and HCl. Work-up that included use of aceto­nitrile solvent led to the formation of a few crystals of the title compound.

## Chemical context   

We have published recently on the synthesis of a series of tetra­methyl­alkyl­cyclo­penta­dienyliridium complexes by the direct reaction between tetra­methyl­alkyl­cyclo­penta­diene and iridium chloride, giving the [Cp*^R^IrCl_2_]_2_ dimer (Morris *et al.*, 2014 ▸). From the dimer, a variety of other compounds can be made, such as amino acid complexes, that have shown significant anti-mycobacterial activity (Karpin *et al.*, 2013 ▸). Some of the reactions produced low yields of the chlorido-bridged dimer, thus limiting the number of products that could be made and tested. 
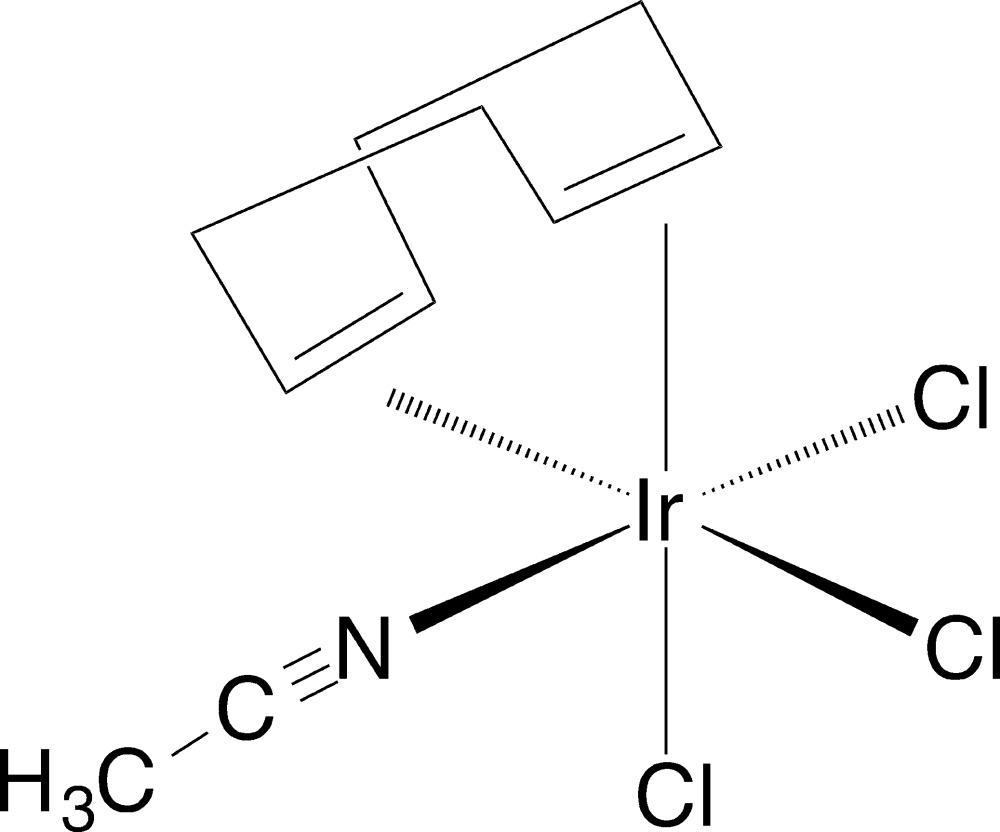


An alternate route to Cp*-type chlorido iridium dimers was reported using [(COD)IrCl]_2_ as the starting material (El Amouri *et al.*, 1994 ▸) and, in our hands, this route does have promise for providing higher yields for many of the compounds. However, in the case of indene, there was no indication that an indenyl iridium complex had been prepared. Instead, a yellow–brown intra­ctable solid was formed. Several attempts to dissolve the solid and to separate products through fractional crystallization all failed. During the course of this work-up, one of the solvents used was aceto­nitrile. At some point, the product mixture was allowed to stand in solution, and after about 24 hours several very nicely shaped rectangular prisms had formed in the sample. These crystals were examined by X-ray crystallography and the results of that structure determination are reported here.

## Structural commentary   

**Figure 1:**
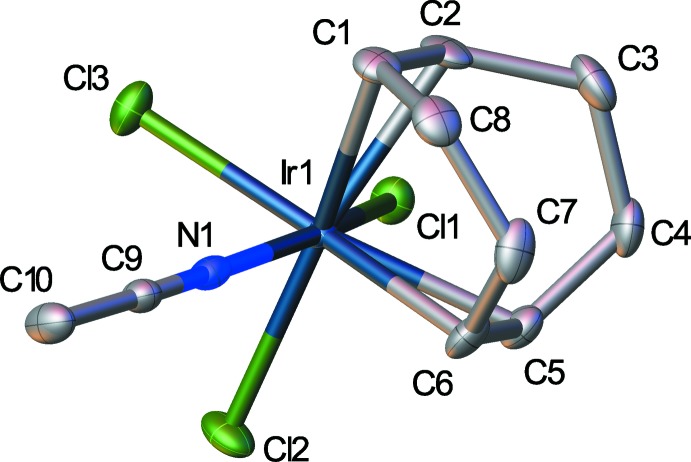
The asymmetric unit of the title compound. Displacement ellipsoids are shown at the 50% probability level.

While the total number of cyclo­octa-1,5-diene complexes structurally characterized is quite large, the number that are directly comparable to the title compound is small. The title compound is a pseudo-octa­hedral complex of iridium with three chloride ligands occupying one face of the octa­hedron and the alkenes of the COD and the aceto­nitrile ligand occupying the opposite face (Fig. 1 ▸). Considering the varying ligands about the central iridium, there is very little distortion from ideal octa­hedral angles, with the most significant distortion being the N1—Ir1—Cl2 distorted away from the COD group with an angle of 164.05 (11)°. All other angles, including those involving the alkene centroids, deviate by no more than 5° from the ideal. All three Ir—Cl bond lengths are similar [range 2.3603 (11) to 2.3670 (11) Å], which is in keeping with both types of *trans* ligands, alkene and aceto­nitrile, being expected to be strong *trans*-influence ligands and would have a similar magnitude of effect on the chloride *trans* to either ligand.

The facial Ir—Cl distances may be contrasted with the average distance of 2.441 (2) Å for *fac*-[(Me_3_P)_3_IrCl_3_] (CCDC: 896073) and related compounds (Merola *et al.*, 2013 ▸) that have somewhat longer Ir—Cl distances due to the effect of the *trans* PMe_3_ groups.

Choudhury *et al.* (2005 ▸) reported on a COD complex of iridium with three chlorides and a SnCl_3_ ligand completing the octa­hedral coordination about the central Ir atom (CCDC: 273475). In that case, though, the compound is a dinuclear one with Ir—Cl—Ir bridges. So, there are long Ir—Cl bonds (those involved in bridging) of 2.544 (4) Å and a shorter terminal Ir—Cl bond of 2.385 (6) Å. C=C bond lengths for the COD ring are similar to the title compound at 1.38 (1) and 1.41 (2) Å.

## Supra­molecular features   

Although there appear to be some close C—H⋯Cl inter­molecular inter­actions, there are no important supra­molecular features to speak of in this structure.

## Database survey   

A substructure search of the CCDC (Groom & Allen, 2014 ▸) for the 1,5-COD-Ir fragment resulted in over 850 hits. This is not a surprising result since [CODIrCl]_2_ is a convenient, high-yield organometallic starting material made in one step from IrCl_3_·H_2_O and cyclo­octa-1,5-diene (Crabtree & Morris, 1977 ▸). From [CODIrCl]_2_, a wide variety of ligand addition, chloride replacement or bridge-splitting reactions can be carried out, leading to a wide variety of compounds containing the COD chelate. Using *Mercury* (Macrae *et al.*, 2008 ▸), an analysis of the COD–Ir search of the database for structures with an octahedral coordination around the metal showed that the C=C bonds of the COD ligands ranged from 1.184 to 1.508 Å with a mean of 1.394 Å. For the title compound, the values of 1.392 (7) and 1.389 (6) Å are pretty much right at the mean for COD C=C bonds.

An analysis of the CCDC database (Groom & Allen, 2014 ▸) for octa­hedral iridium complexes with aceto­nitrile ligands uncovered 99 hits with Ir—N distances measuring from a minimum of 1.897 Å to a maximum of 2.246 Å with a mean of 2.068 Å. For the title compound, the Ir—N distance of 2.023 (4) Å places it just below the mean.

## Synthesis and crystallization   

The title complex was formed as a few isolated crystals from an attempted reaction between [(COD)IrCl]_2_ and indene with HCl in an attempt to synthesize the [indenylIrCl_2_]_2_ dimer, which would have been a useful starting material for our studies. Unfortunately, this did not provide the desired product. The reaction produced some very intra­ctable solids. After multiple attempts to dissolve the solid in many different solvents, including aceto­nitrile, some well-shaped prisms formed on the side of the flask and these crystals were used in this investigation and were shown to be that of the title complex. Attempts to make this material in a rational fashion were not successful.

## Refinement   

Crystal data, data collection and structure refinement details are summarized in Table 1 ▸. H atoms were positioned geometrically and refined as riding with C—H = 0.96–0.98 Å, and with *U*_iso_(H) = 1.2*U*_eq_(C) or 1.5*U*_eq_(C_meth­yl_).

## Supplementary Material

Crystal structure: contains datablock(s) I. DOI: 10.1107/S2056989015004855/pk2547sup1.cif

Structure factors: contains datablock(s) I. DOI: 10.1107/S2056989015004855/pk2547Isup2.hkl

Supporting information file. DOI: 10.1107/S2056989015004855/pk2547Isup3.mol

CCDC reference: 1053035

Additional supporting information:  crystallographic information; 3D view; checkCIF report

## Figures and Tables

**Table 1:** Experimental details

Crystal data	
Chemical formula	[IrCl_3_(C_2_H_3_N)(C_8_H_12_)]
*M*_r_	447.78
Crystal system, space group	Orthorhombic, *P*2_1_2_1_2_1_
Temperature (K)	100
*a*, *b*, *c* ()	8.25131(10), 11.85605(14), 12.94150(15)
*V* (^3^)	1266.04(3)
*Z*	4
Radiation type	Mo *K*
(mm^1^)	11.15
Crystal size (mm)	0.22 0.15 0.11

Data collection	
Diffractometer	Agilent Xcalibur Eos Gemini ultra
Absorption correction	Analytical (*SCALE3 ABSPACK*; Clark Reid, 1995 ▸)
*T*_min_, *T*_max_	0.204, 0.396
No. of measured, independent and observed [*I* > 2(*I*)] reflections	27207, 4333, 4173
*R*_int_	0.040
(sin /)_max_ (^1^)	0.755

Refinement	
*R*[*F* ^2^ > 2(*F* ^2^)], *wR*(*F* ^2^), *S*	0.019, 0.038, 1.08
No. of reflections	4333
No. of parameters	137
H-atom treatment	H-atom parameters constrained
_max_, _min_ (e ^3^)	1.07, 0.74
Absolute structure	Flack *x* determined using 1715 quotients [(*I* ^+^)(*I* )]/[(*I* ^+^)+(*I* )] (Parsons *et al.*, 2013 ▸)
Absolute structure parameter	0.011(4)

Computer programs: *CrysAlis PRO* (Agilent, 2014 ▸), *SHELXS97* and *SHELXL97* (Sheldrick, 2008 ▸) and *OLEX2* (Dolomanov *et al.*, 2009 ▸).

## supplementary crystallographic information

### Crystal data

**Table:**

[IrCl_3_(C_2_H_3_N)(C_8_H_12_)]	*D*_x_ = 2.349 Mg m^−^^3^
*M**_r_* = 447.78	Mo *K*α radiation, λ = 0.71073 Å
Orthorhombic, *P*2_1_2_1_2_1_	Cell parameters from 12030 reflections
*a* = 8.25131 (10) Å	θ = 4.0–32.2°
*b* = 11.85605 (14) Å	µ = 11.15 mm^−^^1^
*c* = 12.94150 (15) Å	*T* = 100 K
*V* = 1266.04 (3) Å^3^	Prism, clear light orange
*Z* = 4	0.22 × 0.15 × 0.11 mm
*F*(000) = 840	

### Data collection

**Table:**

Agilent Xcalibur Eos Gemini ultra diffractometer	4333 independent reflections
Radiation source: Enhance (Mo) X-ray Source, Agilent Gemini System	4173 reflections with *I* > 2σ(*I*)
Graphite monochromator	*R*_int_ = 0.040
Detector resolution: 16.0122 pixels mm^-1^	θ_max_ = 32.4°, θ_min_ = 3.4°
ω scans	*h* = −11→12
Absorption correction: analytical (SCALE3 ABSPACK; Clark & Reid, 1995)	*k* = −17→17
*T*_min_ = 0.204, *T*_max_ = 0.396	*l* = −19→19
27207 measured reflections	

### Refinement

**Table:**

Refinement on *F*^2^	Hydrogen site location: inferred from neighbouring sites
Least-squares matrix: full	H-atom parameters constrained
*R*[*F*^2^ > 2σ(*F*^2^)] = 0.019	*w* = 1/[σ^2^(*F*_o_^2^) + (0.0124*P*)^2^ + 1.2315*P*] where *P* = (*F*_o_^2^ + 2*F*_c_^2^)/3
*wR*(*F*^2^) = 0.038	(Δ/σ)_max_ = 0.002
*S* = 1.08	Δρ_max_ = 1.07 e Å^−^^3^
4333 reflections	Δρ_min_ = −0.74 e Å^−^^3^
137 parameters	Absolute structure: Flack *x* determined using 1715 quotients [(*I*^+^)-(*I*^-^)]/[(*I*^+^)+(*I*^-^)] (Parsons *et al.*, 2013)
0 restraints	Absolute structure parameter: −0.011 (4)
Primary atom site location: structure-invariant direct methods	

### Special details

**Table:**

Geometry. All e.s.d.'s (except the e.s.d. in the dihedral angle between two l.s. planes) are estimated using the full covariance matrix. The cell e.s.d.'s are taken into account individually in the estimation of e.s.d.'s in distances, angles and torsion angles; correlations between e.s.d.'s in cell parameters are only used when they are defined by crystal symmetry. An approximate (isotropic) treatment of cell e.s.d.'s is used for estimating e.s.d.'s involving l.s. planes.

### Fractional atomic coordinates and isotropic or equivalent isotropic displacement parameters (Å^2^)

**Table:**

	*x*	*y*	*z*	*U*_iso_*/*U*_eq_	
Ir1	0.70199 (2)	0.47821 (2)	0.68347 (2)	0.01178 (4)	
Cl1	0.68059 (17)	0.64680 (9)	0.78048 (9)	0.0238 (2)	
Cl2	0.85370 (14)	0.58778 (9)	0.56587 (9)	0.0202 (2)	
Cl3	0.47576 (13)	0.52459 (13)	0.57940 (9)	0.0275 (2)	
N1	0.5308 (5)	0.4220 (3)	0.7828 (3)	0.0153 (7)	
C1	0.7775 (6)	0.3484 (4)	0.5677 (3)	0.0210 (9)	
H1	0.7227	0.3540	0.5008	0.025*	
C2	0.6888 (7)	0.2940 (4)	0.6442 (3)	0.0210 (9)	
H2	0.5813	0.2686	0.6219	0.025*	
C3	0.7669 (7)	0.2197 (4)	0.7247 (4)	0.0268 (12)	
H3A	0.8639	0.1863	0.6953	0.032*	
H3B	0.6930	0.1587	0.7413	0.032*	
C4	0.8132 (6)	0.2806 (4)	0.8253 (4)	0.0245 (9)	
H4A	0.7241	0.2736	0.8737	0.029*	
H4B	0.9063	0.2430	0.8553	0.029*	
C5	0.8528 (5)	0.4040 (4)	0.8121 (4)	0.0202 (9)	
H5	0.8518	0.4477	0.8764	0.024*	
C6	0.9554 (6)	0.4463 (4)	0.7360 (4)	0.0229 (10)	
H6	1.0132	0.5148	0.7568	0.028*	
C7	1.0494 (6)	0.3734 (5)	0.6606 (4)	0.0289 (12)	
H7A	1.0816	0.3048	0.6959	0.035*	
H7B	1.1476	0.4132	0.6414	0.035*	
C8	0.9592 (6)	0.3407 (4)	0.5616 (4)	0.0254 (11)	
H8A	0.9963	0.3892	0.5061	0.030*	
H8B	0.9885	0.2640	0.5437	0.030*	
C9	0.4307 (5)	0.3980 (4)	0.8376 (3)	0.0174 (9)	
C10	0.3017 (7)	0.3700 (4)	0.9097 (3)	0.0246 (9)	
H10A	0.2025	0.4047	0.8874	0.037*	
H10B	0.3294	0.3972	0.9773	0.037*	
H10C	0.2879	0.2896	0.9120	0.037*	

### Atomic displacement parameters (Å^2^)

**Table:**

	*U*^11^	*U*^22^	*U*^33^	*U*^12^	*U*^13^	*U*^23^
Ir1	0.01257 (6)	0.01427 (6)	0.00849 (6)	0.00042 (6)	0.00024 (6)	0.00067 (6)
Cl1	0.0356 (7)	0.0166 (5)	0.0193 (5)	−0.0010 (5)	0.0073 (5)	−0.0029 (4)
Cl2	0.0229 (5)	0.0206 (5)	0.0170 (5)	−0.0021 (4)	0.0046 (4)	0.0034 (4)
Cl3	0.0177 (5)	0.0456 (7)	0.0191 (5)	0.0062 (5)	−0.0037 (4)	0.0099 (6)
N1	0.0216 (19)	0.0134 (17)	0.0110 (16)	−0.0012 (14)	−0.0036 (14)	0.0013 (13)
C1	0.027 (3)	0.022 (2)	0.014 (2)	−0.002 (2)	0.0019 (19)	−0.0038 (16)
C2	0.031 (2)	0.0162 (19)	0.0162 (19)	−0.006 (2)	0.002 (2)	−0.0054 (15)
C3	0.041 (3)	0.016 (2)	0.024 (2)	0.007 (2)	0.012 (2)	0.0051 (18)
C4	0.026 (2)	0.027 (2)	0.020 (2)	0.0060 (19)	−0.001 (2)	0.0097 (19)
C5	0.0196 (19)	0.028 (2)	0.0133 (19)	0.0018 (16)	−0.0060 (19)	0.006 (2)
C6	0.015 (2)	0.036 (3)	0.018 (2)	0.0004 (18)	−0.0079 (17)	0.0053 (19)
C7	0.016 (2)	0.040 (3)	0.030 (3)	0.010 (2)	0.0045 (19)	0.009 (2)
C8	0.030 (3)	0.024 (2)	0.022 (2)	0.008 (2)	0.011 (2)	−0.002 (2)
C9	0.0157 (19)	0.021 (2)	0.015 (2)	−0.0023 (16)	−0.0014 (15)	−0.0035 (16)
C10	0.020 (2)	0.037 (3)	0.017 (2)	−0.007 (2)	0.003 (2)	−0.0031 (18)

### Geometric parameters (Å, º)

**Table:**

Ir1—Cl1	2.3670 (11)	C4—H4A	0.9700
Ir1—Cl2	2.3603 (11)	C4—H4B	0.9700
Ir1—Cl3	2.3666 (10)	C4—C5	1.509 (6)
Ir1—N1	2.023 (4)	C5—H5	0.9800
Ir1—C1	2.236 (4)	C5—C6	1.392 (7)
Ir1—C2	2.245 (4)	C6—H6	0.9800
Ir1—C5	2.257 (5)	C6—C7	1.517 (7)
Ir1—C6	2.231 (4)	C7—H7A	0.9700
N1—C9	1.125 (6)	C7—H7B	0.9700
C1—H1	0.9800	C7—C8	1.531 (8)
C1—C2	1.389 (6)	C8—H8A	0.9700
C1—C8	1.504 (7)	C8—H8B	0.9700
C2—H2	0.9800	C9—C10	1.454 (6)
C2—C3	1.510 (7)	C10—H10A	0.9600
C3—H3A	0.9700	C10—H10B	0.9600
C3—H3B	0.9700	C10—H10C	0.9600
C3—C4	1.537 (7)		
			
Cl2—Ir1—Cl1	85.23 (4)	C2—C3—H3B	108.6
Cl2—Ir1—Cl3	85.61 (4)	C2—C3—C4	114.6 (4)
Cl3—Ir1—Cl1	92.68 (5)	H3A—C3—H3B	107.6
N1—Ir1—Cl1	83.63 (11)	C4—C3—H3A	108.6
N1—Ir1—Cl2	164.05 (11)	C4—C3—H3B	108.6
N1—Ir1—Cl3	83.55 (11)	C3—C4—H4A	108.6
N1—Ir1—C1	113.18 (16)	C3—C4—H4B	108.6
N1—Ir1—C2	77.86 (16)	H4A—C4—H4B	107.6
N1—Ir1—C5	77.73 (16)	C5—C4—C3	114.5 (4)
N1—Ir1—C6	113.88 (16)	C5—C4—H4A	108.6
C1—Ir1—Cl1	163.18 (12)	C5—C4—H4B	108.6
C1—Ir1—Cl2	78.41 (12)	Ir1—C5—H5	114.5
C1—Ir1—Cl3	89.92 (14)	C4—C5—Ir1	110.0 (3)
C1—Ir1—C2	36.12 (16)	C4—C5—H5	114.5
C1—Ir1—C5	94.14 (18)	C6—C5—Ir1	70.9 (3)
C2—Ir1—Cl1	159.73 (12)	C6—C5—C4	124.1 (5)
C2—Ir1—Cl2	114.52 (12)	C6—C5—H5	114.5
C2—Ir1—Cl3	93.37 (14)	Ir1—C6—H6	113.6
C2—Ir1—C5	79.33 (18)	C5—C6—Ir1	73.0 (3)
C5—Ir1—Cl1	88.79 (13)	C5—C6—H6	113.6
C5—Ir1—Cl2	113.44 (12)	C5—C6—C7	124.2 (5)
C5—Ir1—Cl3	160.95 (12)	C7—C6—Ir1	112.4 (3)
C6—Ir1—Cl1	92.97 (14)	C7—C6—H6	113.6
C6—Ir1—Cl2	78.03 (13)	C6—C7—H7A	108.4
C6—Ir1—Cl3	162.18 (12)	C6—C7—H7B	108.4
C6—Ir1—C1	79.97 (19)	C6—C7—C8	115.7 (4)
C6—Ir1—C2	87.1 (2)	H7A—C7—H7B	107.4
C6—Ir1—C5	36.15 (17)	C8—C7—H7A	108.4
C9—N1—Ir1	175.1 (4)	C8—C7—H7B	108.4
Ir1—C1—H1	114.7	C1—C8—C7	115.2 (4)
C2—C1—Ir1	72.3 (3)	C1—C8—H8A	108.5
C2—C1—H1	114.7	C1—C8—H8B	108.5
C2—C1—C8	122.3 (5)	C7—C8—H8A	108.5
C8—C1—Ir1	110.8 (3)	C7—C8—H8B	108.5
C8—C1—H1	114.7	H8A—C8—H8B	107.5
Ir1—C2—H2	114.2	N1—C9—C10	178.4 (5)
C1—C2—Ir1	71.6 (3)	C9—C10—H10A	109.5
C1—C2—H2	114.2	C9—C10—H10B	109.5
C1—C2—C3	122.5 (5)	C9—C10—H10C	109.5
C3—C2—Ir1	113.0 (3)	H10A—C10—H10B	109.5
C3—C2—H2	114.2	H10A—C10—H10C	109.5
C2—C3—H3A	108.6	H10B—C10—H10C	109.5
			
Ir1—C1—C2—C3	−106.1 (4)	C3—C4—C5—Ir1	32.7 (5)
Ir1—C1—C8—C7	27.6 (5)	C3—C4—C5—C6	−47.3 (6)
Ir1—C2—C3—C4	10.1 (6)	C4—C5—C6—Ir1	101.7 (4)
Ir1—C5—C6—C7	−105.8 (4)	C4—C5—C6—C7	−4.2 (7)
Ir1—C6—C7—C8	3.1 (6)	C5—C6—C7—C8	87.1 (6)
C1—C2—C3—C4	92.3 (5)	C6—C7—C8—C1	−20.8 (7)
C2—C1—C8—C7	−54.0 (6)	C8—C1—C2—Ir1	103.8 (4)
C2—C3—C4—C5	−29.0 (6)	C8—C1—C2—C3	−2.3 (7)
